# Lipid peroxidation and immune activation: TRAF3’s double-edged strategy against glioblastoma

**DOI:** 10.1172/JCI190471

**Published:** 2025-04-01

**Authors:** Tzu-Yi Chia, Nishanth S. Sadagopan, Jason Miska

**Affiliations:** 1 Department of Neurological Surgery, and; 2 Malnati Brain Tumor Institute of the Lurie Comprehensive Cancer Center, Feinberg School of Medicine, Northwestern University, Chicago Illinois, USA.

## Abstract

Glioblastoma (GBM), the most aggressive type of primary brain tumor, continues to defy therapeutic advances with its metabolic adaptability and resistance to treatment. In this issue of the *JCI*, Zeng et al. delve into a pivotal mechanism underpinning this adaptability. They identified an important role for TNF receptor–associated factor 3 (TRAF3) in regulating lipid metabolism through its interaction with enoyl-CoA hydratase 1 (ECH1). These findings elucidate a unique signaling axis that shields GBM cells from lipid peroxidation and antitumor immunity, advancing therapeutic strategies for GBM that may also carry over to other cancers with similar metabolic vulnerabilities.

## Unpacking the metabolic landscape of glioblastoma

Glioblastoma (GBM), the most aggressive form of primary brain cancer, remains a formidable therapeutic challenge because of its metabolic adaptability and resistance to conventional treatments (1). GBM exploits its metabolic plasticity to thrive in nutrient-depleted and hypoxic microenvironments, reprogramming cellular pathways to sustain growth and evade immune surveillance. Lipids, essential for building cell membranes, are indispensable for cellular growth. However, lipid metabolism has emerged as a hallmark of GBM pathophysiology, with many studies revealing how GBM alters fatty acid oxidation (FAO) as a source of ATP to support its malignancy (2, 3). What makes the metabolic demands of brain tumors unique from other malignancies is the limited availability of free fatty acids (FAs) in the CNS compared with the peripheral tissues (3). Consequently, GBM cells depend on mechanisms such as producing their own lipids or receiving them from myelin-phagocytizing macrophages to sustain their malignant process (4, 5). This CNS-specific metabolic environment creates unique dependencies in GBM, differentiating its lipid metabolism from other malignancies and offering novel therapeutic targets.

A crucial subset of these lipids, polyunsaturated fatty acids (PUFAs), cannot be synthesized de novo and must either be recycled or obtained from dietary sources in the CNS (6). Unique to the CNS is the overabundance of arachidonic acid (AA) and docosahexaenoic acid (DHA), which are essential for normal brain development and function (7). While these PUFAs are advantageous for malignancy due to their roles in maintaining membrane fluidity and cellular signaling, their presence also poses a metabolic liability because of their susceptibility to oxidative damage through lipid peroxidation (8). Moreover, the relative abundance of membrane PUFAs, monounsaturated fatty acids (MUFAs), and saturated fatty acids (SFAs) is indicative of susceptibility to ferroptosis, a form of cell death driven by lipid peroxidation (9). Thus, in the CNS, tumor growth opportunities and metabolic vulnerabilities create unique dependencies that can be exploited for antitumor treatments. In this issue of the *JCI*, Zeng et al. identified TRAF3 as a critical modulator of PUFA metabolism and immune resistance in GBM (10). By targeting the TNF receptor–associated factor 3/enoyl-CoA hydratase 1 (TRAF3/ECH1) axis, they revealed an innovative approach to disrupt tumor metabolism while enhancing antitumor immunity (10).

## The role of TRAF3 in GBM

TRAF3 is a member of the TNF receptor–associated (TNFR-associated) factor family, best known for its roles in innate immune signaling, scaffolding function, and E3 ubiquitin ligase activities (11). Canonically, TRAF3 typically controls the inflammatory activation of immune cells. For example, a deficiency of TRAF3 in humans impairs TLR3 signaling and prevents the production of type 1 IFN in response to infection (12). Conversely, TRAF3 is a key negative regulator of the alternative NF-κB pathway, ubiquitinating NF-κB–inducing kinase (NIK) to limit its activation (13). While the functions of TRAF3 in immune cells, such as T and B lymphocytes, are well documented, its roles in cancer, particularly in GBM, have been underexplored (11). Zeng et al. (10) revealed that TRAF3 was frequently repressed in GBM as a result of promoter hypermethylation, a common epigenetic alteration in cancer that silences tumor suppressor genes (10, 14). Poor survival outcomes were correlated with lower expression of TRAF3 in patients with GBM (10). This mirrors other hypermethylation phenotypes that influence antitumor immunity in GBM. For example, the stimulator of interferon gene (STING) promoter in GBM is also hypermethylated, dampening IFN-mediated antitumor immunity (15). Thus, hypermethylation of immune-modulatory genes may be a common mechanism by which GBM protects itself from immune attack.

Through overexpression of TRAF3, Zeng and authors demonstrated the ability of TRAF3 to reduce FA oxidation (FAO) activity and induce ROS production and mitochondrial damage in GBM cells. By interacting with and ubiquitinating ECH1, TRAF3 disrupted the mitochondrial localization of ECH1, impeding its role in PUFA oxidation. This action promoted the accumulation of peroxidized lipids, thereby enhancing oxidative stress and sensitizing GBM cells to ferroptosis (9). These findings position TRAF3 as a key regulator of lipid metabolism in GBM tumorigenesis (10).

However, caution is warranted when interpreting the FAO data. The experiments supporting this aspect of the study used etomoxir at a high enough concentration known to inhibit mitochondrial complex I, disrupt CoA metabolism, and induce severe oxidative stress (10, 16–18). Since these off-target effects occur independently of CPT1-mediated FAO, they represent a limitation of the proposed mechanisms of this study. (10).

## The TRAF3/ECH1 axis provides molecular insights

**Figure 1:**
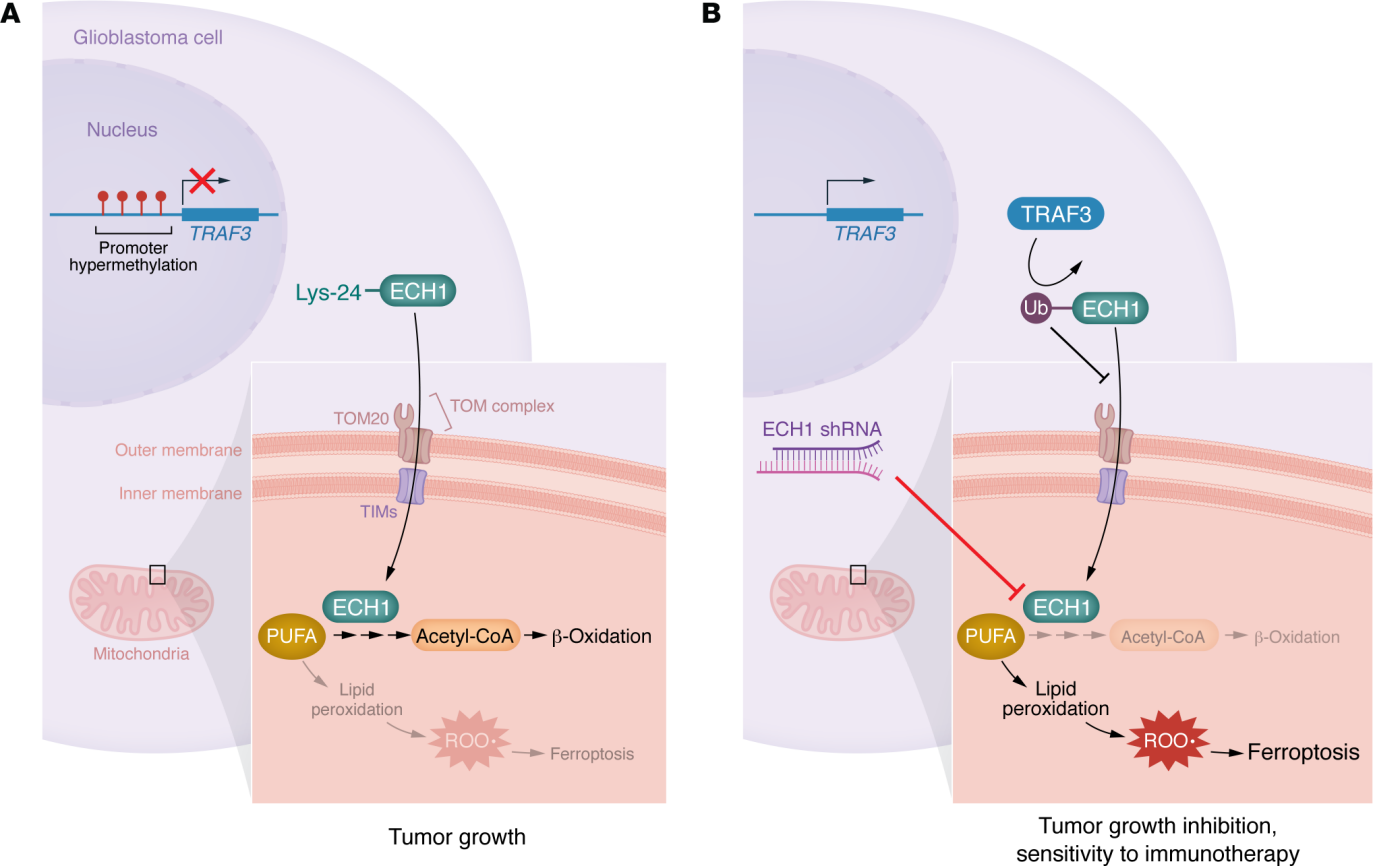
TRAF3 has a regulatory role in GBM via PUFA metabolism and its hypermethylation. (**A**) In the context of GBM, promoter hypermethylation suppresses TRAF3 expression. The absence of TRAF3 permits ECH1-mediated metabolism of PUFAs and efficient FAO. (**B**) Zeng et al. showed that TRAF3 overexpression resulted in the ubiquitination (Ub) of ECH1, which impeded FAO, promoted lipid peroxidation, and induced ferroptosis. Furthermore, ECH1 depletion via shRNA induced mitochondrial damage and inhibited tumorigenesis. These findings underscore the therapeutic potential of targeting hypermethylation and the TRAF3/ECH1 axis to suppress tumor growth and enhance sensitivity to immunotherapy.

Zeng et al. (10) provide detailed molecular insights into the TRAF3/ECH1 axis. ECH1, a mitochondrial enzyme, played a crucial role in the isomerization of unsaturated FAs (UFAs), a prerequisite if their fate is mitochondrial β-oxidation. The authors identified TRAF3 as a mediator of ECH1 ubiquitination at Lys214 via K63-linked chains, a modification that disrupted its interaction with the mitochondrial translocase TOMM20 and prevented its translocation into the mitochondria. ECH1 inhibition led to the accumulation of lipids instead of their catabolization by β-oxidation, thereby shifting the balance toward lipid peroxidation and proferroptotic responses. Notably, TRAF3 ubiquitination of ECH1 disrupted its mitochondrial localization, shifting lipid metabolism toward peroxidation and ferroptosis (Figure 1) (10). Additionally, T cells are well known to potentiate tumor killing through ferroptosis (19). Therefore, ECH1 inhibition could potentially enhance the efficacy of immune checkpoint blockade therapies. Indeed, the authors demonstrated that overexpression of TRAF3 increased sensitivity to programmed death–ligand 1 (PD-L1) blockade in murine models of GBM (10).

Importantly, TRAF3’s tumor-suppressive effects were independent of its canonical role in modulating NF-κB signaling (20). Instead, an NF-κB–independent mechanism occurred, whereby TRAF3 regulated metabolic plasticity and oxidative stress through the ubiquitination of ECH1 in GBM (10). This finding expands our understanding of TRAF3’s multifaceted roles in glioma progression.

## Therapeutic implications

The therapeutic implications of the TRAF3/ECH1 axis are profound and offer an alternative therapeutic approach by exploiting the metabolic vulnerability of GBM. By restoring TRAF3 expression, Zeng et al. (10) achieved marked increases in lipid peroxidation, oxidative stress, and ferroptosis sensitivity in GBM cells. As CD8^+^ T cells mediate tumor cell killing through ferroptosis, synergizing the reprogramming of lipid metabolism with anti–PD-L1 immunotherapy enhances the immune system’s ability to eliminate tumor cells (10, 21). The results of Zeng et al. (10) highlight the dual potential of TRAF3 targeting: disrupting the metabolic defenses of GBM while amplifying immune-mediated cytotoxicity. Combining TRAF3 restoration with ferroptosis inducers or checkpoint inhibitors may offer synergistic benefits, as oxidative stress sensitizes tumor cells to immune-mediated killing.

Given that TRAF3 silencing in GBM is largely driven by promoter hypermethylation, a viable therapeutic strategy may involve using demethylating agents, such as DNA methyltransferase inhibitors (DNMTis), to restore TRAF3 expression. Epigenetic reprogramming could reestablish immune sensitivity while simultaneously enhancing ferroptotic vulnerability, offering a promising dual-targeting approach. For example, previous work has shown that decitabine (a DMNMTi) treatment “turns on” new neoantigens and embryonic antigens in GBM, enhancing T cell–mediated cytotoxicity of GBM (22). This approach was also show with regard to STING activation; treatment with decitabine could derepress STING promoter methylation, increasing its activation and promoting antitumor immunity (16). Thus, Zeng et al. (10) identify yet another way that epigenetic targeting may be a useful strategy to enhance immunotherapeutic responses to GBM.

However, translating the TRAF3/ECH1 axis into clinical application presents several challenges. Efficient gene delivery systems are essential to prevent off-target effects and ensure sustained expression levels to overcome the epigenetic repression of TRAF3 in tumors. Nanoparticle-based delivery platforms, which have shown promise in RNA and protein therapeutics, may offer a viable solution (23). However, nanoparticle-based delivery faces challenges such as limited bioavailability, potential immunogenicity, and the need for tumor-specific targeting to avoid off-target effects.

Regarding the use of epigenetic inhibitors, the problems with this strategy are potentially manifold. For example, DNMTis have severe off-target effects, resulting in dose-limiting toxicities (primarily hepatocellular), and can induce cytopenias, which may last for months after treatment (24). Additionally, the overall response rate of DNMTis is rather low, especially for solid tumors. However, several ongoing and developing combinatorial approaches, including immunotherapies, are being explored to determine whether DNMTis can boost antitumor immune responses (25). Therefore, the study by Zeng et al. (10) provides further evidence that these inhibitors may serve as immune-stimulatory agents.

## Unanswered questions and future directions

The Zeng et al. (10) findings raise several compelling questions that warrant further exploration. One important avenue of inquiry lies in understanding how the TRAF3/ECH1 axis integrates with other metabolic pathways, such as glycolysis and lipid synthesis, which are critical for GBM proliferation and survival. The interplay between these pathways may reveal additional vulnerabilities that could be targeted therapeutically. Furthermore, the implications of TRAF3-mediated lipid peroxidation for the tumor microenvironment remain poorly understood. Lipid metabolism byproducts could influence immune cell function, potentially modulating the efficacy of immune checkpoint blockade and other immunotherapies. This work not only advances GBM therapeutics but may also inform strategies for other cancers with similar metabolic vulnerabilities, potentially broadening the impact of these findings.

Another important consideration is whether the metabolic vulnerabilities associated with TRAF3 restoration are unique to GBM or extend to other cancers with similar reliance on PUFA metabolism. Investigating these questions could not only refine therapeutic strategies for GBM but also broaden the applicability of these findings to other malignancies. Finally, the clinical translation of TRAF3-targeting strategies will require addressing challenges such as efficient delivery mechanisms, specificity, and sustained activity. Advances in nanotechnology and targeted delivery systems offer promising avenues to overcome these hurdles, paving the way for innovative combination therapies that exploit the metabolic and immune vulnerabilities of GBM.

## Conclusion

Zeng et al. (10) illuminate a critical metabolic checkpoint in GBM, linking the regulatory role of TRAF3 to PUFA metabolism and immune resistance. Their findings not only enhance our understanding of the metabolic landscape of GBM but also open new avenues for therapeutic intervention. The integration of metabolic and immune-targeting approaches — leveraging insights into the TRAF3/ECH1 axis—holds promise for improving outcomes in this challenging malignancy. As research progresses, these discoveries could herald a new era of precision medicine for GBM and other metabolically driven cancers.

## Version 1 04/01/2025

Electronic publication
